# Supplementary material for: The causal role of circulating inflammatory markers in osteoporosis: a bidirectional Mendelian randomized study
Source: Front Immunol. 2024 Jul 18;15:1412298. doi: 10.3389/fimmu.2024.1412298 (PMC11291241; doi:10.3389/fimmu.2024.1412298)
Supplement: Supplementary file 1 [file DataSheet_1.zip › supplementary material/OSTEOPOROSIS-CIM/GCST90274831.tsv.gz_leave_one_out.pdf]

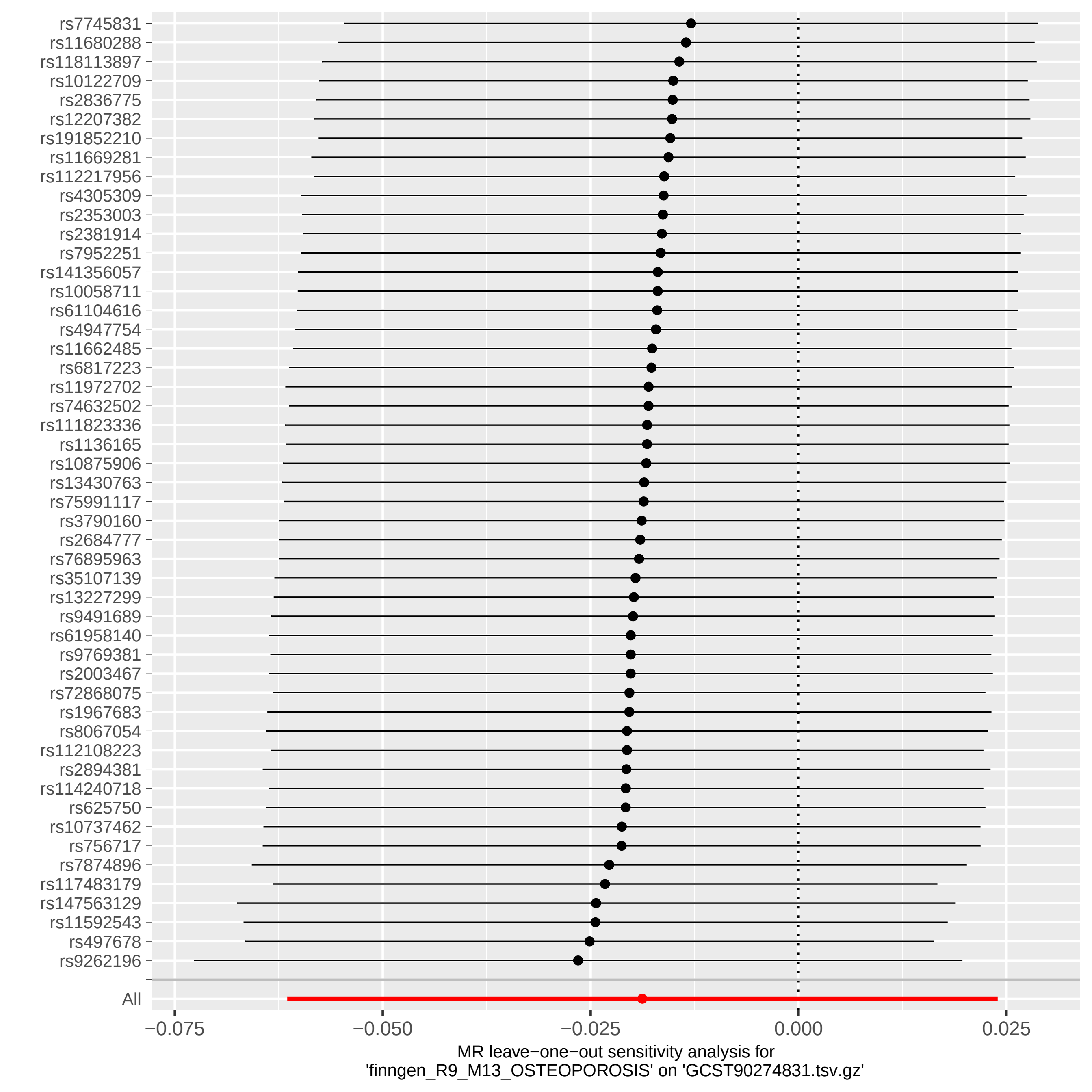

rs7745831
rs11680288
rs118113897
rs10122709
rs2836775
rs12207382
rs191852210
rs11669281
rs112217956
rs4305309
rs2353003
rs2381914
rs7952251
rs141356057
rs10058711
rs61104616
rs4947754
rs11662485
rs6817223
rs11972702
rs74632502
rs111823336
rs1136165
rs10875906
rs13430763
rs75991117
rs3790160
rs2684777
rs76895963
rs35107139
rs13227299
rs9491689
rs61958140
rs9769381
rs2003467
rs72868075
rs1967683
rs8067054
rs112108223
rs2894381
rs114240718
rs625750
rs10737462
rs756717
rs7874896
rs117483179
rs147563129
rs11592543
rs497678
rs9262196
All
−0.075
−0.050
−0.025
0.000
0.025
MR leave−one−out sensitivity analysis for
'finngen_R9_M13_OSTEOPOROSIS' on 'GCST90274831.tsv.gz'
